# Inhibition of NSUN3 suppresses immune escape in non-small cell lung cancer through stabilizing PD-L1 in a 5-methyladenosine dependent way

**DOI:** 10.1016/j.clinsp.2025.100662

**Published:** 2025-04-24

**Authors:** Hancong Huang, Xiaohong Lv, Qianhua Chen, Lixia Dong

**Affiliations:** a Department of Respiratory and Critical Care Medicine, Aviation General Hospital, Beijing, PR China; b Department of Respiratory and Critical Care Medicine, Tianjin Medical University General Hospital, Tianjin, PR China

**Keywords:** Non-small cell lung cancer, NSUN3, Programmed Cell Death 1 ligand 1, CD8-positive T-lymphocytes

## Abstract

**Background:**

Alterations in 5-methyladenosine (m^5^C) RNA methyltransferase have been implicated in the pathogenesis of lung cancer. However, the role of NSUN3, a key m^5^C regulator, in modulating PD-L1 expression and immune evasion in Non-Small Cell Lung Cancer (NSCLC) remains poorly understood.

**Methods:**

NSUN3 expression levels in NSCLC tissues and cell lines were quantified using quantitative Reverse Transcription PCR (RT-qPCR). The impact of NSUN3 expression modulation on lung cancer cell viability and proliferation was assessed through CCK-8 assays and colony formation experiments. To evaluate immune cell interactions, CD8+ *T*-cells were co-cultured with NSCLC cells, and cytotoxic activity was measured by an LDH cytotoxicity detection kit. The in vivo tumorigenic role of NSUN3 was investigated using subcutaneous tumor xenograft models. Mechanistic insights into NSUN3-PD-L1 interactions were obtained through m^5^C-RIP, RIP, and dual-luciferase reporter assays.

**Results:**

Elevated NSUN3 mRNA levels were observed in NSCLC tissues compared to normal controls. Functional studies demonstrated that NSUN3 knockdown significantly inhibited cell viability and proliferation in both A549 and PC9 cell lines. Furthermore, NSUN3 depletion enhanced CD8+ *T*-cell-mediated cytotoxicity against NSCLC cells and suppressed tumor growth in vivo. Mechanistically, NSUN3 inhibition was found to stabilize PD-L1 mRNA, suggesting a regulatory role in immune checkpoint modulation.

**Conclusions:**

The present findings establish NSUN3 as an oncogenic driver in NSCLC pathogenesis, functioning through PD-L1 mRNA stabilization. The identification of this NSUN3-PD-L1 regulatory axis provides new insights into the molecular mechanisms underlying immune escape in NSCLC and offers promising directions for developing targeted immunotherapies.

## Highlights

• NSUN3 levels were up-regulated in NSCLC.
• Inhibition of NSUN3 promotes CD8+ *T*-cell cytotoxicity against NSCLC cells.
• NSUN3 inhibition suppresses the tumor growth.

## Introduction

Lung cancer has the highest incidence and mortality of malignant tumors in the world, of which Non-Small Cell Lung Cancer (NSCLC) accounts for about 80 % and has a low 5-year survival rate,^1^ In recent years, molecular targeted therapy for lung cancer has become increasingly mature in clinical application, among which Epidermal Growth Factor Receptor Tyrosine Kinase Inhibitors (EGFR-TKI) have been the most widely used, making a breakthrough in the treatment of NSCLC.^2^ However, almost all patients were gradually insensitive to EGFR-TKI after treatment for a period of time (the median time was 9 to 14 months), resulting in acquired drug resistance, and a small number of patients carrying sensitive mutations were insensitive to the drug at the initial treatment stage, that is, primary drug resistance.^3^ Therefore, finding new targets for NSCLC therapy and synergistic anticancer has become a research focus.

Epigenetic modification mainly includes DNA methylation, RNA methylation and histone modification. These epigenetic regulations play a crucial role in the occurrence and development of cancers.^4^ At the same time, many studies have shown that epigenetic modification changes also occur in the process of tumor drug resistance.^5^ In recent years, RNA methylation modification, represented by 5-methyladenosine (m^5^C), has become a frontier and hot spot in epigenetics research.

RNA m^5^C modification plays a critical role in the pathogenesis of NSCLC. For instance, RNA m^5^C hypermethylation and NSUN2 (a m^5^C writer) were significantly correlated with intrinsic resistance to EGFR-TKI.^6^ NSUN2 also enhances the stability of NRF2 mRNA through the m^5^C modification within its 5′UTR region to regulate ferroptosis in NSCLC.^7^ By blocking the interaction of T-cell surface Programmed cell Death Protein 1 (PD1) with its ligands Programmed cell Death 1 Ligand 1 (PD-L1) and Programmed cell Death 1 Ligand 2 (PD-L2), the inhibition of immune microenvironment can be eliminated, and the ability of T-cells to kill non-small cell lung cancer can be enhanced.^8^^,^^9^ NSUN2 was also confirmed to positively regulate the expression of PD-L1 in lung cancer cells and may be a potential prognostic biomarker related to immunity in NSCLC.^10^ Therefore, the study of m^5^C modification of PDL1 is of reference significance for the study of inhibiting immune escape in NSCLC.

Studies have shown that m^5^C regulators can predict the clinical prognostic risk of lung cancer and regulate the tumor immune microenvironment.^11^, ^12^, ^13^ The expressions of two m^5^C regulators, NSUN3 and NSUN4, were significantly higher in lung cancer tissues than in normal groups, and this increase is associated with clinicopathological features and survival.^14^ In addition, NSUN3 and NSUN4 are related to the infiltration of six major immune cells, especially NSUN3 is closely related to CD8+ *T*-cells.^14^ However, whether NSUN3 regulates PDL1 in an m^5^C dependent manner in NSCLC remains unclear and warrants further investigation.

## Materials and methods

### Clinical sample collection

This study was performed in line with the principles of the Declaration of Helsinki. Approval was granted by the Ethics Committee of Aviation General Hospital (Approval No. 2019005). Informed consent was obtained from all individual participants included in the study. All methods were carried out in accordance with relevant guidelines and regulations. Twenty-two cases of NSCLC tissue specimens and corresponding adjacent ones from the hospital were collected and stored in liquid nitrogen for use. All tissue samples were verified by pathological examination. Patients signed informed consent, and this study was approved by the hospital.

### Cell culture

Human NSCLC cell lines (A549 and PC9) obtained from Beyotime Company and human normal bronchial epithelial cell line HBE obtained from ATCC were cultured in DMEM supplemented with 10 % fetal bovine serum (FBS; Gibco) and 1 % penicillin/streptomycin (Beyotime). All cells were free from mycoplasma contamination and kept in a humidified atmosphere containing 5 % CO_2_ at 37 °C.

### Isolation of peripheral blood mononuclear cells (PBMCs) and CD8+ *T*-cells

Blood samples were collected and analyzed from the individual healthy volunteers (*n* = 9) in the hospital. The procedure for PBMCs isolation was the same as described previously.^15^ The CD8+ *T*-cell isolation kit (Miltenyi Biotec) was used to isolate CD8+ *T*-cells from the PBMCs under the guidance of the manufacturer's manual.

### Cell viability, cytotoxicity, and proliferation evaluation

CCK-8 assay (Sigma) was used to evaluate the cell viability of lung cancer cells according to the manufacturer's protocol. In brief, 1 × 10^3^ lung cancer cells were seeded in a 96-well plate, whereas a four-times repeat was necessary for measuring the cell viability in a time-dependent manner. The CCK-8 reagent was added 72 h after cell transfection, and the cells were further incubated at 37 °C for 4 h and absorbance was measured at 450 nm on a microplate reader (Thermo Fisher). CD8+ *T*-cells were incubated with lung cancer cells for 48 h, and the cytotoxic effect of CD8+ *T*-cells on lung cancer cells was determined using the LDH cytotoxicity detection kit (Thermo Fisher). Cell proliferation activity was measured by staining with the Cell-Light EdU Apollo567 In Vitro Kit (Ribobio) according to the manufacturer's instruction and further analyzed on fluorescence microscopy. As for the cell colony formation assay, cells seeded into the 6-well plates (1000 cells/well) were stained with 0.1 % crystal violet (Beyotime) for 1 h. Cell number was counted using a gel documentation system (BioRad).

### Quantitative reverse transcription PCR (RT-qPCR)

RT-qPCR was used to detect mRNA levels of NSUN3 and PDL1 in both tissues and cells. Briefly, RNA in tissues or cells was extracted using TRIzol reagent (Invitrogen), A cDNA synthesis kit purchased from Thermo Fisher was used for reverse transcription. All PCR reactions were performed on 7900 fast real-time detection with the TaqMan RT-PCR method (Applied Biosystems). GAPDH acted as the endogenous control, and the 2^-ΔΔCT^ method was for transcript expression level measurement. The primer sequences were used as follows: NSUN3: Forward 5′-CATGCTGGCAATATGCTGTCC-3′, Reverse 5′-AAAGATCCCTGAGAGAGTGTGT-3′; PD-L1: Forward 5′-TGGCATTTGCTGAACGCATTT-3′, Reverse 5′-TGCAGCCAGGTCTAATTGTTTT-3′; GAPDH: Forward 5′-GGAGCGAGATCCCTCCAAAAT-3′, Reverse 5′-GGCTGTTGTCATACTTCTCATGG-3′.

### Western blot assay

Protein was extracted from tissues and cells using the RIPA lysis buffer (Beyotime), and separated by 10 % SDS-PAGE electrophoresis, and transferred to the polyvinylidene difluoride membranes (Millipore), followed by the incubation with indicating primary antibodies and peroxidase-conjugated secondary antibody, respectively. Subsequently, the protein bands were visualized by chemiluminescence and quantified by the Image J software. The antibodies used in this experiment were as follows: anti-NSUN3 (ab272616, 1:300, Abcam), anti-PDL1 (ab205921, 1:300, Abcam), and anti-β-actin (ab8227, 1:800, Abcam).

### Bioinformatic analysis

RNAm^5^C finder (http://www.rnanut.net/rnam5cfinder/), A sequence-based m^5^C modification site predictor, was used to predict the potential m^5^C modification site of PD-L1.

### RNA immunoprecipitation (RIP)

The RIP assay was performed using a Magna RIP® RIP Kit (17–700, Millipore) according to the manufacturer's instructions. Briefly, the supernatant of tissue homogenate was collected after RIPA lysate treatment and centrifugation (14,000 rpm, 4 °C). Then the supernatant was taken as input and then was incubated with the antibody for precipitation. The samples and input were detached with proteinase K (10 mg/mL) to extract RNA for subsequent qPCR detection of target RNA. The antibodies used were as follows: anti-NSUN3 (ab272616, 1:30, Abcam), IgG (10,285–1-AP, Proteintech).

### m^5^C-RIP

Purified mRNA was fragmented into around 100-nucleotide-long fragments using RNA Fragmentation Reagents (Invitrogen, AM8740). About 400 ng of fragmented mRNAs were mixed with 2.5 µg of anti-m^5^C antibody (ab214727, Abcam) in immunoprecipitation buffer and incubated by rotating at 25 °C for 1 h. The mixture was then immunoprecipitated by incubation with prewashed Protein A Magnetic Beads (Thermo Scientific, 10002D) at 4 °C for 5 h. After extensive washing, the bound RNA fragments were eluted from the beads by proteinase K digestion at 55 °C for 60 min. Finally, RNA was isolated from the eluate by phenol-chloroform extraction and ethanol plus glycogen (Thermo Scientific, AM9516) precipitation for qRT-PCR analysis.

### Double luciferase reporter gene experiment

The wild-type fragments of PD-L1 were constructed and named PD-L1-WT. The wild-type gene fragments were amplified and inserted into the pmirGLO dual-luciferase reporter gene vector using endonuclease sites Spe I and Hind III. The complementary sequence mutation sites of the seed sequences were designed on the wild-type fragments of PD-L1-WT, named PD-L1-MUT, and the mutant gene fragments were amplified by restriction endonuclease and T4 DNA ligase inserted into the pmirGLO vector. These reporter plasmids and shNC or shNSUN2 were co-transfected into 293T cells using Lipofectamine3000 reagent. After 48 h, the cells were collected and fully lysed. After centrifugation, the supernatant was collected and treated with the luciferase reaction reagent. The cell lysate was then added, and the firefly luciferase activity was finally measured and normalized to the renin luciferase activity.

### Subcutaneous tumor xenograft models

The 6-week-old male BALB/c nude mice were fed for 1 week. The 8 × 10^6^ A549 cells transfected with different plasmids were digested with pancreatic enzyme, centrifugally washed with precooled PBS twice, suspended with precooled PBS, and subcutically inoculated into the armpits of nude mice. The long and short diameters of the tumors were measured with vernier-caliper three times a week. Tumor volume = long diameter × short diameter^2^ × 0.5. Four weeks later, the tumor tissue was dissected, and the tumor's body weight was weighed. The tumor tissues were fixed with 4 % paraformaldehyde, embedded in paraffin, sliced, and stained with Hematoxylin-Eosin (HE) to observe the histopathological changes of the tumor. Tumor tissues removed from mice were incubated with anti-NSUN3 antibody or anti-PD-L1 antibody at 4 °C overnight. The expression of NSUN3/PD-L1 was analyzed based on the color and staining intensity.

### Statistical analysis

The experimental data were analyzed by GraphPad Prism software version 8.3, and the data operation between the two groups was represented by mean ± SD and compared with a *t*-test. One-way ANOVA was used to compare the mean of multiple groups. Tukey′s post hoc test was used to compare pairwise comparisons between groups. The p-value less than 0.05 means the difference is statistically significant.

## Results

### NSUN3 inhibition promotes CD8+ *T*-cell cytotoxicity against NSCLC cells and tumor growth

**Fig. 1:**
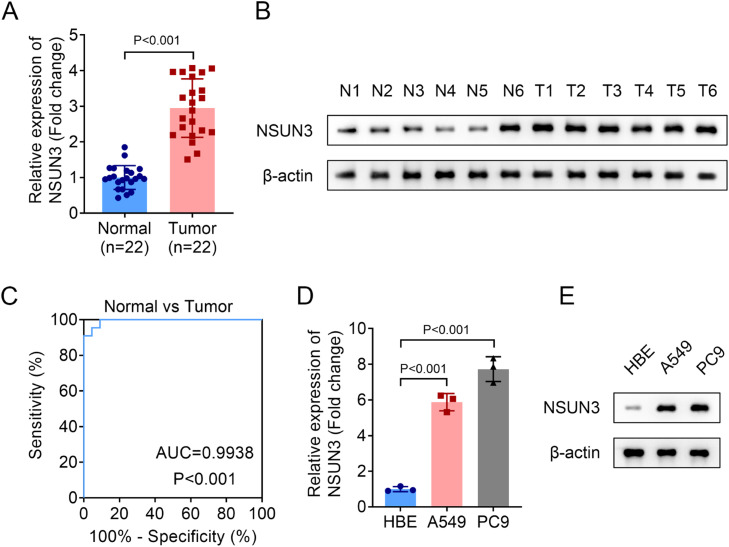
NSUN3 levels were upregulated in NSCLC. (A) Quantitative histogram of NSUN3 mRNA levels in NSCLC tissues and normal lung tissues from qPCR analysis (*n* = 22). (B) Representative protein images which represent the NSUN3 protein levels in six paired clinical samples with NSCLC and adjacent tissues analyzed by western blot (*n* = 3). (C) The ROC curve of NSUN3 in NSCLC. (D) Relative mRNA expression of NSUN3 in human normal bronchial epithelial cell line HBE and NSCLC cell lines (A549 and PC9) from qPCR analysis (*n* = 3). (E) Protein levels of NSUN3 in HBE, A549 and PC9 cells are determined by western blot (*n* = 3).

**Fig. 2:**
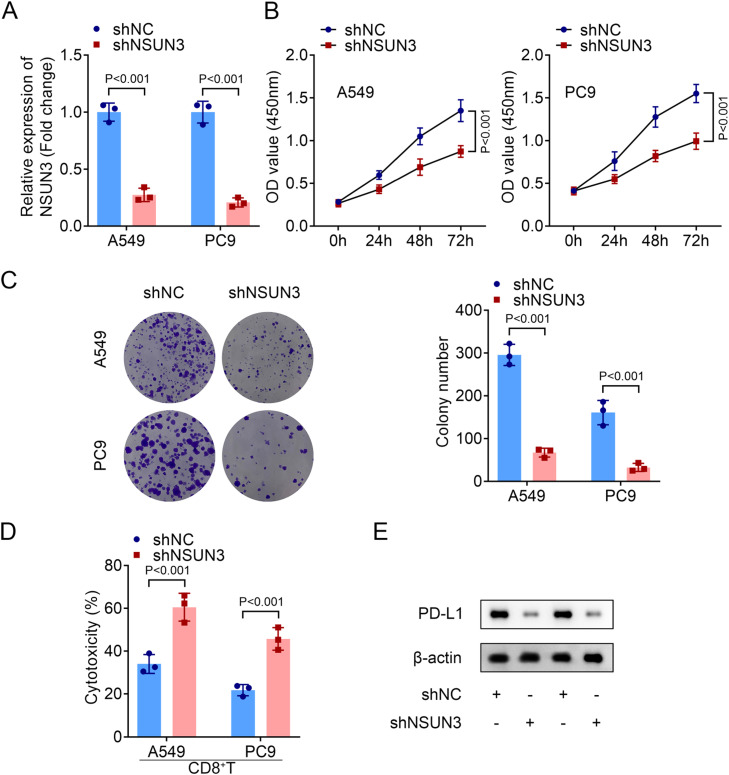
Inhibition of NSUN3 promotes CD8+ *T*-cell cytotoxicity against NSCLC cells. (A) Relative mRNA levels of NSUN3 in NSCLC cells after transfection are determined by q-PCR (*n* = 3). (B) Cell viability of NSCLC cells before and after transfection is evaluated by CCK-8 assay (*n* = 3). (C) Cell proliferation of NSCLC cells before and after transfection is evaluated by colony formation assay (*n* = 3). (D) Cytotoxicity of CD8+ *T*-cells is detected via LDH cytotoxicity assay (*n* = 3). (E) Protein levels of PD-L1 in NSCLC cells before and after transfection are determined by western blot (*n* = 3).

**Fig. 3:**
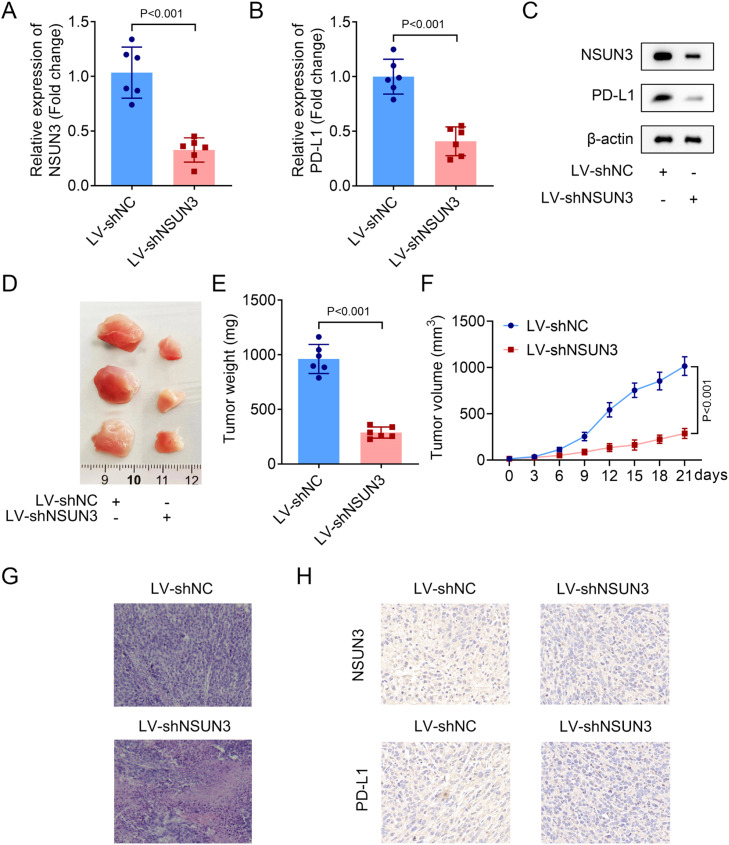
NSUN3 inhibition suppresses the tumor growth. (A‒B) Relative mRNA levels of NSUN3 and PD-L1 in the exfoliated tumors of subcutaneous tumor xenograft models are determined by q-PCR (*n* = 6). (A) Protein levels of NSUN3 and PD-L1 in the exfoliated tumor tissues were determined by western blot (*n* = 3). (B) Representative images of the exfoliated tumors of subcutaneous tumor xenograft models. (E‒F) The tumor weight and volume of the exfoliated tumors of subcutaneous tumor xenograft models, *n* = 6, *p* < 0.001. (G) Representative images of the exfoliated tumor tissues after HE-staining (*n* = 3). (H) Protein levels of NSUN3 and PD-L1 in the exfoliated tumor tissues were determined by immunohistochemistry (*n* = 3).

Firstly, the relative expression of NSUN3 in tumor tissues of NSCLC was evaluated, and both the mRNA and protein levels of NSUN3 in tumor groups were significantly higher than those in normal groups according to the analysis of qPCR and western blot methods (Fig. 1A‒B). Meanwhile, the results of ROC suggested that the AUC is 0.9938 with *p* < 0.001, indicating that highly expressed NSUN3 has high sensitivity and specificity in NSUN3 (Fig. 1C). Subsequently, the relative expression of NSUN3 in NSCLC cell lines was also be detected. As indicated in Fig. 1D and Fig. 1E, NSUN3 levels were prominently elevated in both A549 and PC9 cells compared with HBE normal cells (Fig. 1D‒E). Based on the data that NSUN3 is highly expressed in NSCLC, the authors wondered whether NSUN3 is involved in the regulation of NSCLC cells in vitro. Cell viability and proliferation of both A549 and PC9 cells were evaluated before and after the successful knockdown of NSUN3 using cell transfection technology. qPCR analysis demonstrated that the stably lowly expressed NSUN3 NSCLC cells were constructed (Fig. 2A). The results of CCK-8 assay suggested that inhibition of NSUN3 significantly suppressed the cell viability of both A549 and PC9 cells (Fig. 2B). The proliferation of both A549 and PC9 cells were also been inhibited by NSUN3 inhibition (Fig. 2C). Down-regulation of NSUN3 prominently promoted the cytotoxicity of CD8+ *T*-cells against NSCLC cells (Fig. 2D). PDL1 protein expression was also downregulated by the inhibition of NSUN3 (Fig. 2E). After injection of lentivirus packaging inhibition NSUN3 vectors, both NSUN3 and PD-L1 levels were significantly down-regulated (Fig. 3A‒C). The size of exfoliated tumors was measured, and the results suggested that the size of the tumors in LV-shNSUN3 group was visibly reduced compared with the LV-shNC group (Fig. 3D). Additionally, the tumor weight (Fig. 3E) and tumor volume (Fig. 3F) of LV-shNSUN3 were also significantly lower than those in LV-shNC group. Down-regulation of NSUN3 significantly alleviated inflammatory infiltration of tissues according to the results of the HE-staining method (Fig. 3G). The immunohistochemical results suggested that NSUN3 knockdown could decrease the expression of both NSUN3 and PD-L1 (Fig. 3H).

### NSUN3 stabilized the mRNA of PD-L1 in an m5C dependent way

**Fig. 4:**
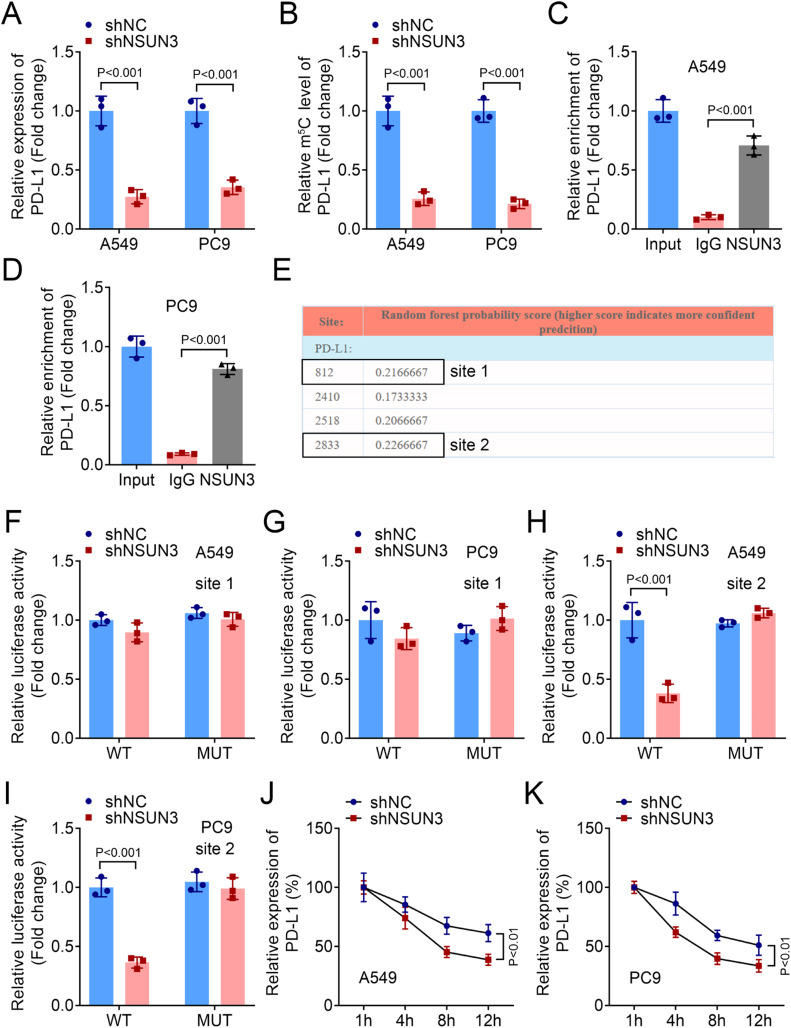
NSUN3 stabilized mRNA of PD-L1 in a m^5^C dependent way. (A) Quantitative histogram of PD-L1 mRNA levels in A549 and PC9 cells from qPCR analysis (*n* = 3). (B) The interaction between PD-L1 and m^5^C antibody was verified by m^5^C-RIP method (*n* = 3). (C‒D) The interaction between PD-L1 and NSUN2 antibody was verified by RIP method (*n* = 3). (E) The potential m^5^C modification sites of PD-L1 predicted by RNAm^5^C finder. (F‒I) Dual luciferase reporter assay is performed to evaluate the binding of PD-L1 and NSUN3 (*n* = 3). (J‒K) qPCR analysis indicates the levels of PD-L1 expression in NSCLC cells treated with actinomycin D (2 μg/mL) at the indicated time points (*n* = 3).

After NSUN3 was down-regulated in A549 and PC9 cells, PD-L1 levels were also down-regulated (Fig. 4A). Therefore, the authors speculated that PD-L1 may be m^5^C modified mediated by NSUN3 in macrophages. The m^5^C-RIP assay was implemented to assess the m5C modification status of PD-L1 mRNA. The results exhibited that the level of PD-L1 can be enriched by m^5^C antibody, and inhibition of NSUN3 down-regulated the m^5^C medicated level of PD-L1 (Fig. 4B). RIP followed by qPCR found that compared with IgG antibody, NSUN3 antibody significantly enriched PD-L1 (Fig. 4C‒D). Subsequently, the potential m^5^C modification sites of PD-L1 predicted by RNAm^5^C finder suggested that PD-L1 may be m^5^C modified at two sites (Fig. 4E). After mutation at each of the two sites, the results of the double-luciferase gene report experiment suggested that there was no significant change in relative luciferase activity before and after NSUN3 inhibition at mutation sites 1, but after mutation at site 2, down-regulation of NSUN3 could significantly reduce relative luciferase activity (Fig. 4F‒I). Moreover, inhibition of NSUN3 promoted the degradation of PD-L1 mRNA (Fig. 4J‒K).

## Discussion

In recent decades, the primary treatment methods for patients with advanced lung cancer have been radiotherapy and chemotherapy. However, their efficacy remains limited, and they are often associated with significant toxic side effects. PD-1, an important immunosuppressive molecule, plays a critical role in tumor immune evasion.^16^ By binding with PD-L1, PD-1 can block T-cell receptor signaling and co-stimulatory pathways, thereby inhibiting T-cell activation and proliferation, ultimately allowing tumor cells to escape immune surveillance. As a key immune checkpoint, PD-1 and its ligands have been extensively studied in the field of cancer immunotherapy. Current knowledge and possibilities in immunotherapy targeting PD-1 and PD-L1 have been reviewed and summarized in recent studies.^17^, ^18^, ^19^

The stabilization of PD-L1 mRNA, modulated by various epigenetic regulations, has been reported in multiple cancers.^20^ For instance, the METTL3/IGF2BP3 axis-mediated stabilization of PD-L1 mRNA suppresses anti-tumor immunity in breast cancer.^21^ Similarly, the JNK/METTL3 axis influences PD-L1-mediated T-cell activation, exhaustion, and infiltration in bladder cancer.^22^ In NSCLC, elevated levels of circIGF2BP3, regulated by PD-L1, are negatively correlated with CD8+ *T*-cell infiltration. Furthermore, METTL3 mediates the m^6^A modification of circIGF2BP3, highlighting the intricate interplay between RNA modifications and immune regulation in cancer.^23^

The Tumor Immune Microenvironment (TIME) is closely linked to tumor progression and the response to immunotherapy. Recent studies have shown that m^5^C modification can regulate the infiltrating immune cells in tumors.^24^, ^25^, ^26^ Multiple m^5^C regulatory proteins in TIME have been identified as prognostic and diagnostic markers of cancers.^24^, ^25^, ^26^ For example, in lung adenocarcinoma, patients with a high m^5^C level had a better prognosis, and different m^5^C modifications represent different immune infiltration.^27^ Specifically, the high m^5^C modification levels are positively correlated with neutrophils, resting CD4+ memory T-cells, and M2 macrophages in lung squamous cell carcinoma, and negatively correlated with follicular helper T-cells, CD8+ *T*-cells, and activated NK cells.^28^

NSUN2, a m^5^C RNA methyltransferase, plays a pivotal role in the methylation of tRNA and mRNA.^29^ Its abnormal expression has been linked to the development and progression of various tumors.^30^^,^^31^ For example, NSUN2 is involved in GRB2-mediated colon cancer cell migration. GRB2-associated binding protein, as the targeted regulatory gene of miR-125b, is a typical pro-tumor cell migration protein. The high expression of NSUN2 in colorectal cancer inhibits miR-125b and thus promotes the expression of GRB2-associated binding protein, resulting in an enhanced migration rate of cancer cells.^32^^,^^33^ These findings highlight the importance of m5C modifications in regulating tumor biology and immune responses.

Recently, a seven-gene m^6^A/m^5^C risk model, comprising METTL3, NPLOC4, RBM15, YTHDF1, IGF2BP1, NSUN3, and NSUN7, was developed to stratify the prognosis of early-stage lung cancer.^34^ Among these genes, NSUN3 is a protein-coding gene with RNA binding and methyltransferase activities. It has been reported that NSUN3-mediated m^5^C modification of mitochondrial tRNA enhances energy supply by promoting protein synthesis in the mitochondrial respiratory chain, thereby promoting cancer cell invasion and metastasis.^35^ In this study, the authors found that NSUN3 was up-regulated in NSCLC, and its inhibition suppressed cell viability and proliferation. Importantly, down-regulation of NSUN3 prominently reduced PD-L1 levels and enhanced the cytotoxicity of CD8+ *T*-cells against NSCLC cells. These findings align with the role of NSUN3 in liver hepatocellular carcinoma, where it similarly promotes tumor immune escape.^36^

The authors further investigated the mechanism by which NSUN3 promotes tumor immune evasion and found that NSUN3 enhances the stability of PD-L1 through tumor endogenous mechanisms, primarily via m^5^C modification. This regulation underscores the potential of targeting NSUN3 and m^5^C modifications to improve immunotherapy outcomes in NSCLC. Overall, the present findings contribute to a growing body of evidence highlighting the critical role of RNA modifications in shaping the tumor immune microenvironment and suggest novel therapeutic avenues for lung cancer treatment.

## Conclusion

In this study, for the first time, the regulatory role of NSUN3 in the body's anti-tumor immune effect was discovered and its mechanism was initially discussed. It was proposed that inhibition of NSUN3 could inhibit the expression of PD-L1 in NSCLC cells by binding to PD-L1. The discovery that NSUN3 is involved in the regulation of tumor immunity in NSCLC, and provides ideas for the subsequent anti-tumor target drug design and the development of new tumor immunotherapy strategies.

## Abbreviations

m5C, 5-methyladenosine; NSCLC, Non-Small Cell Lung Cancer; RT-qPCR, quantitative Reverse Transcription PCR; EGFR-TKI, Epidermal Growth Factor Receptor Tyrosine Kinase Inhibitors; FBS, Fetal Bovine Serum; PBMCs, Peripheral Blood Mononuclear Cells; TIME, Tumor Immune Microenvironment; PD1, Programmed cell Death protein 1; PD-L1, Programmed cell Death 1 ligand 1; PD-L2, Programmed cell Death 1 ligand 2.

## Ethics approval and consent to participate

This study was approved by the Ethics Committee of Aviation General Hospital (Approval No. 2019005). This study was performed in line with the principles of the Declaration of Helsinki. Informed consent was obtained from all individual participants included in the study. All animal experiments should comply with the ARRIVE guidelines. All methods were carried out in accordance with relevant guidelines and regulations.

## Consent for publication

Not applicable.

## Availability of data and materials

The datasets used and/or analyzed during the current study are available from the corresponding author upon reasonable request.

## Authors’ contributions

All authors participated in the design, interpretation of the studies and analysis of the data, and review of the manuscript. H.H. and X.L. drafted the work and revised it critically for important intellectual content; Q.C. was responsible for the acquisition, analysis, and interpretation of data for the work; L.D. made substantial contributions to the conception or design of the work. All authors read and approved the final manuscript.

## Funding

The authors declare that no funds, grants, or other support were received during the preparation of this manuscript.

## Declaration of competing interest

The authors declare no conflicts of interest.
